# Microplastics prevalence in water, sediment and two economically important species of fish in an urban riverine system in Ghana

**DOI:** 10.1371/journal.pone.0263196

**Published:** 2022-02-03

**Authors:** Emmanuel R. Blankson, Patricia Nakie Tetteh, Prince Oppong, Francis Gbogbo

**Affiliations:** Department of Animal Biology and Conservation Science, University of Ghana, Accra, Ghana; Tsinghua University, CHINA

## Abstract

Urban riverine systems serve as conduits for the transport of plastic waste from the terrestrial dumpsites to marine repositories. This study presented data on the occurrence of microplastics in water, sediment, Bagrid Catfish (*Chrysichthys nigrodigitatus)* and Black-chinned Tilapia (*Sarotherodon melanotheron*) from the Densu River, an urban riverine system in Ghana. Microplastics were extracted from the samples collected from both the lentic and lotic sections of the river. The results indicated widespread pollution of the Densu River with microplastics in all the compartments studied. The average numbers of microplastic particles deposited in the Dam (2.0 ± 0.58) and Delta (2.50 ± 0.48) sections of the river were not affected by the differences in their hydrology. However, the stagnant water system of the Dam promoted the floating of larger-sized microplastics while the flowing waters of the Delta did not show any selectivity in the deposition of microplastics between sediment and the water column. The number of microplastics ingestions by the Bagrid Catfish (2.88 ± 2.11) was similar to the Black-chinned Tilapia (2.38 ± 1.66) but both species ingested lower numbers of microplastics than reported for marine fish species in coastal Ghana.

## 1. Introduction

Plastics are one of the most used materials in the world due to their durability, versatility and cost-efficiency. Global production of plastic has therefore been increasing over the years with about 360 million tons of plastic produced in 2018 [1]. As a result of the increased usage of plastic, plastic wastes have become a global environmental problem and about five (5) to 13 million tons of plastic wastes are discharged annually into the marine environment [2]. Plastics degrade into smaller particles of less than 5 mm known as microplastics, and less than 0.1 μm, nanoplastics, that are ubiquitous. The deleterious effects of microplastics are enormous and include causing physical damage or injury to a variety of exposed aquatic organisms and the release of Persistent Organic Pollutants upon ingestion [3–5]. Microplastics are therefore a threat to aquatic life and food webs.

In the 20^th^ century, the most problematic contaminants in Africa comprised those from the petroleum and agricultural industries and include tarballs, nutrient runoff, and coastal sediment fouling from a host of land-based industries [6]. In recent times, African countries are experiencing major challenges with the control of plastic wastes with five African countries (Egypt, Nigeria, South Africa, Algeria and Morocco) among the top 20 plastic waste producers in the world [7]. This notwithstanding, a review of current literature assessed microplastic research in Africa to be at its infancy largely due to the lack of capacity of institutions [8].

In Ghana, over 3000 tons of plastic wastes are generated daily while over 250,000 tons are dumped into the Atlantic Ocean annually [9, 10]. It is estimated that as much as 85% of the plastic wastes generated throughout the country is made up of single used water sachets and an estimated 8.2 billion plastic water sachets are consumed annually in Ghana. Many plastic wastes in Ghana are transported to the marine system by rivers traversing human settlements which at the same time, serve as a source of fisheries for local communities. Microplastic ingestion by fish and other organisms is a well-known phenomenon. Besides the deleterious physical effects of microplastic on biota, plastics contain wide ecotoxic chemicals such as dioxins, persistent organic pollutants and polychlorinated biphenyls that pose additional consequences to biota, human health, and the functioning of ecosystems [11]. Therefore, the importance of investigating microplastic in urban rivers and biota together with the implications of these to human health cannot be overemphasized.

Fish contributes about 60% of Ghana’s animal protein intake [12] but there is limited research on the ingestion of microplastic by fish species in Ghanaian waters. The limited studies on microplastic in Ghana have generally focused on sediment [13], water [14] and marine fish species [15]. Information on microplastic in the riverine ecosystems many of which serve as a conduit for the transport of plastic waste from the terrestrial dump sites to the marine repositories is particularly limited.

The Densu River is one of the important urban rivers in Ghana that traverses several towns. It has 17 species of fish [16] of which the Black-chinned Tilapia (*Sarotherodon melanotheron*) and Bagrid Catfish (*Chrysichthys nigrodigitatus)* are one of the most economically important. The Black-chinned Tilapia is an omnivorous pelagic species foraging principally on phytoplankton, macrophytes, insects, detritus and zooplankton [17] compared to Bagrid Catfish, an omnivorous demersal species foraging on fish, invertebrates, aquatic plants and fish eggs. The Densu River further has two sections at its lower end namely the Weija Dam and the Densu Delta. These two ends experience different water flow regimes with the Dam site holding stagnant water while in the Delta, free river water flows into the sea. Thus, besides the significance of evaluating plastic pollution in the Densu River as an urban riverine system, the Densu River further offers the opportunity to study the effect of two hydrological regimes on the fate and spatial distribution of microplastics. In this study, we report for the first time, data on the occurrence of microplastic in water, sediment and two species of fish from the Densu River, as well as the effect of the two flow regimes in sections of the Densu River on the fate and spatial distribution of microplastics.

## 2. Materials and methods

### 2.1. Study area

The Densu River is 116 km long, arising from the Atewa Range (6.1667° N, 0.6000° W) in the Eastern Region of Ghana. It traverses several towns including Koforidua, Nsawam and Akwadum, Adoagyiri, and Weija and ends in the ecologically significant Densu Delta Ramsar site at the edge of the Atlantic Ocean. The river was dammed at Weija (5.5697° N, 0.3442° W). about 8 km upstream of the Densu Delta (5.5302° N, 0.2902° W). The Weija Dam provides potable water to western parts of Accra-Tema metropolitan areas and offers facilities for irrigation and fishing. At maximum water level, the Weija Dam is 14 km long, 2.2 km wide and has a total surface area of 33.6 km^2^ with a mean depth of 5 m [16]. The Densu Delta on the other hand covers an area of about 50 km^2^ and comprises the saltpans, dunes, flood plains and the lowest part of the Densu River. It is a home for about 60 species of waterbirds with an estimated maximum number of 35,000 birds [18].

The Densu River Basin has about 200 human settlements with a total population of over 600,000, equivalent to 240 persons per km^2^ [19]. Although the river supplies drinking water to the capital city of Ghana, it also receives solid and liquid wastes from the basin settlements including parts of Accra and Kasoa communities. The main economic activities in the catchment area are fishing, animal rearing and crop farming. The Densu Delta is estimated to produce an annual fish yield of 270 tonnes [18].

### 2.2. Processing of fish and microplastic extraction

Fish samples were purchased from fishermen at Weija Dam and Densu Delta between February and April 2021. For each of the sites, 12 specimens of Black-chinned Tilapia and 12 specimens of Bagrid Catfish were purchased given a total of 24 specimens each of Black-chinned Tilapia and Bagrid Catfish and 48 specimens altogether. The purchase of the fish specimens was done using a random sampling approach where the fishermen were selected randomly and a maximum of two fishes were purchased from each selected fisherman. The specimens were transported on ice to the Department of Animal Biology and Conservation Science, University of Ghana and kept frozen until they were ready for analysis.

Each fish specimen was washed with distilled water and body morphometrics taken. The body morphometrics include Total Length which was determined on a fish measuring board while Body Weight was taken with digital weighing balance [20]. The measurements were followed by a dissection of each fish from the anal opening to the head region and the removal of the entire gastrointestinal tracts. The gastrointestinal tract of each fish was digested in Potassium hydroxide (KOH) at 60°C for 24 h as described by [21]. The digested materials were filtered through 1.2 μm Whatman GF/C microfiber filter papers and residues dried at 60°C for 24 h.

### 2.3. Processing and extraction of microplastics in surface water

Surface water samples were collected from three different locations in each of the study habitats into acid-washed glass jars and stored at a temperature of 4°C until analysis. Each of the glass jars of water was thoroughly shaken after which two (2) replicates of 10 ml subsamples were taken, given a total of 12 subsamples altogether. Each 10 ml subsample was then digested with Potassium hydroxide (KOH) at 60°C for 24 h. The digested materials were filtered through 1.2 μm Whatman GF/C microfiber filter papers and residues dried at 60°C for 24 h.

### 2.4. Processing and extraction of microplastics in sediment

Sediment samples were collected from three different locations on each of the study habitats. The sediment samples were separately wrapped in aluminum foils and transported to the laboratory where they were oven-dried at 60°C to constant weight. The dry sediment samples were homogenized with ceramic pestle and mortar and approximately 10 g of each sample was weighed in a glass beaker and mixed with NaCl solution (density ρ = 1.2g/mL) containing a drop of olive oil meant to enable microplastics to gather rather than sticking to the glass walls [14]. Each mixture was stirred for 10 minutes and left for four (4) hours after which the supernatant was slowly poured into glass tubes and digested with Potassium hydroxide (KOH) at 60°C for 24 h.

### 2.5. Identification of microplastics

Microplastics were observed under Leica EZ4 HD stereo microscope with image analyses system IC80 HD camera. The microplastics were counted and their sizes consisting of the length of their longest axis were measured following protocols outlined in the Spotter’s Guide for identifying microplastics in Fish developed by the Civic Laboratory for Environmental Action Research [22].

### 2.6. Data analysis

The data were checked for normality using the Shapiro-Wilk test. Data sets that were not normal were transformed or analyzed using the appropriate non-parametric test. Data were pooled for the two study sites and the differences in size and number of MP between the Black-chinned Tilapia and the Bagrid Catfish were analyzed without consideration of the sites. Additionally, the size and number of MP for the water, sediment, Bagrid Catfish and Black-chinned Tilapia were also analyzed within and between the Weija Dam and Densu Delta. The students’ t-test was used to evaluate differences in the number and size of MP in the water and sediments, as well the number and size of MP in the gastrointestinal (GI) tract of the Black-chinned Tilapia and Bagrid Catfish. The condition factor (K) and growth coefficient (b) were estimated from the length and weight relationship of the fish. The equation for the condition factor and growth coefficient is K = (100*W/L^b^) [23].

The condition factor (K) reflects the physiological state of the fish population and indicates whether the fish is in good use of its resources [20]. An estimated K greater than one (1) indicates good growth conditions and less than one (1) indicates poor growth conditions. Also, the value of the growth coefficient (b) of fish is 3 and indicates isometric growth for fish [24]. A calculated growth coefficient of value less than 3 indicates negative allometric growth and greater than 3 indicates positive allometric growth [24].

## 3. Result

### 3.1. General morphometrics, growth and condition of fish

Table 1 shows the morphometric characteristics, growth coefficients and condition factors of the Bagrid Catfish and Black-chinned Tilapia used in the present study. The length of the Black-chinned Tilapia in the Densu Delta and the Weija Dam ranged from 8.7cm to 19.1cm and 8.6 to 20.1 cm respectively. Similarly, the length of the Bagrid Catfish ranged from 24 cm to 43 cm and 27cm to 49 cm respectively in the Densu Delta and the Weija Dam. With respect to weight, the Black-chinned Tilapia from the Densu Delta and the Weija Dam ranged from 11.86 g to 199.03 g and 11.67 g to 130.98 g respectively. The Bagrid Catfish weighed between 106.7 g to 555 g and 210.4 g to 451.1 g respectively in the Densu Delta and the Weija Dam.

**Table 1:** Morphometric, growth coefficients and condition factors of Bagrid Catfish and Black-chinned Tilapia specimens from Densu Delta and Weija Dam.

Species	n	Site	Length (cm)	Weight (g)	b	K	MP/individual
Black-chinned Tilapia	12	Weija Dam	14.4 ± 4.09	63.68 ± 43.90	2.91	1.77	2.50
Bagrid Catfish	12	Weija Dam	36.32 ± 5.69	358.63 ± 75.27	1.06	0.79	1.58
Black-chinned Tilapia	12	Densu Delta	14.38 ± 3.75	68.93 ± 54.02	3.15	1.87	2.25
Bagrid Catfish	12	Densu Delta	30.68 ± 7.11	262.48 ± 180.50	2.87	0.80	4.17
Black-chinned Tilapia	24	Combined	14.39 ± 3.76	66.31 ± 47.20	3.02	1.83	2.38
Bagrid Catfish	24	Combined	33.50 ± 6.78	310.55 ± 140.86	2.35	0.80	1.58

Analysis of the length data of the fishes indicated no significant difference (P >0.05) in the length of Black-chinned Tilapia from the Densu Delta (14.38 ± 3.75) and the Weija Dam (14.4 ± 4.09). There was however a significant difference (T = 2.15, P = 0.043) in the length of Bagrid Catfish from the Densu Delta (30.68 ± 7.11) and the Weija Dam (36.32 ± 5.69) with a 1.2-fold increase in the length of Bagrid Catfish from the Weija Dam compared to those from the Densu Delta. With respect to weight, the weight of the Black-chinned Tilapia from the Densu Delta (68.93 ± 54.02) and the Weija Dam (63.68 ± 43.90) was not significantly different (P >0.05). Also, there was no significant difference (P >0.05) in the weight of Bagrid Catfish from the Densu Delta (262.48 ± 180.50) and the Weija Dam (358.63 ± 75.27).

The condition factor and growth coefficient of the Black-chinned Tilapia population in the Densu Delta and Weija Dam indicate they were in good conditions and there is isometric growth (Table 1). In contrast, the condition factor for the Bagrid Catfish populations in the Densu Delta (0.8) and Weija dam (0.79) indicates poor conditions. Although the Bagrid Catfish in the present study had poor growth conditions, there was isometric growth for the Bagrid Catfish population in the Densu Delta (2.89) whiles those in the Weija dam showed negative allometric growth (1.06).

### 3.2. Number of microplastic particles in water and sediments

**Fig 1:**
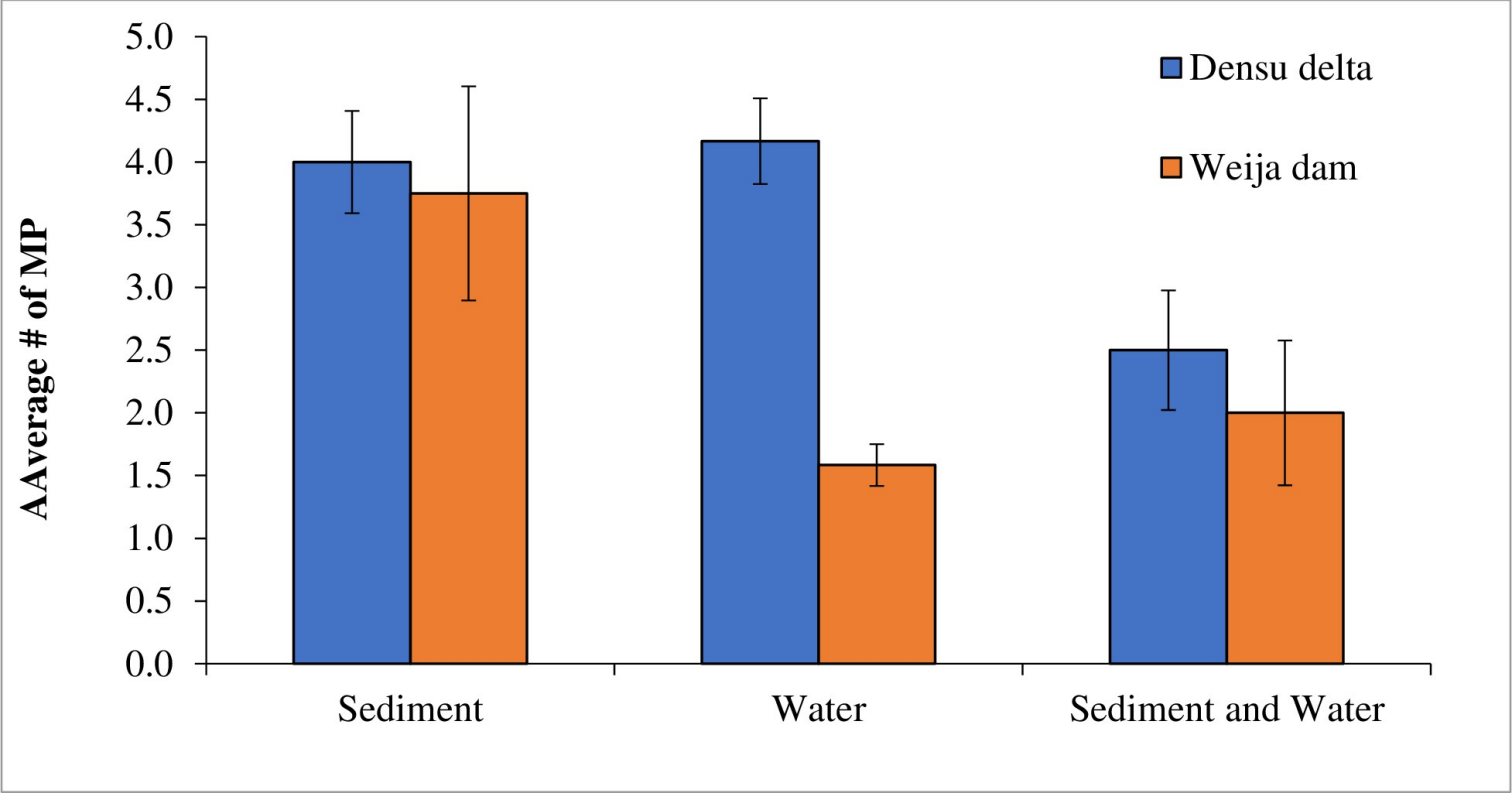
Number of microplastics in sediment and water of the Densu Delta and Weija Dam in Ghana.

A summary of the number of microplastic particles in the various environmental samples collected from the two study habitats is presented in Table 2. Overall, 16 particles of microplastics were recovered per 40 g of sediment from Weija compared to 15 particles of microplastics per 40 g of sediment from Densu Delta. Similarly, 9 particles of microplastics were observed per 60 ml of water from the Weija Dam compared to 5 microplastic particles per 60 ml of water from the Densu Delta. The mean number of microplastics did not differ significantly (U = 10, P = 0.230) between the water samples in the Weija Dam (1.583± 0.167) and Densu Delta (4.16± 0.342) (Fig 1). There was also no significant (T = 0.264, P = 0.801) difference in the mean number of microplastic between the sediment samples in the Weija Dam (3.75± 0.853) and Densu Delta (4.00± 0.408) (Fig 1). With respect to the mean number of microplastics for combined sediment and water in the Weija Dam (2.00± 0.577) and Densu Delta (2.50± 0.477), there was no significant difference (U = 38.5, P = 0.40) (Fig 1).

**Table 2:** Summary data on number of microplastics in sediment, water, Black-chinned Tilapia and Bagrid Catfish from Densu Delta and Weija Dam in Ghana.

Sample type	Site					
	Densu		Weija		Combine	
	Total number	Mean (±SD)	Total number	Mean (±SD)	Total number	Mean (±SD)
Sediment	16 MP per 40g	4 .0 ± 0.82 per 10 g	15 MP per 40g	3.75 ± 1.71 per 10 g	31 MP per 80g	3.88 ± 1.25 per 10 g
Water	9 MP per 60ml	1.5 ± 0.84 per 10 ml	5 MP per 60ml	0.83 ± 0.41 per 10 ml	14 MP per 120 ml	1 ± 0.72 per 10 ml
Black-chinned Tilapia	27 MP per 12 gut	2.67 ± 1.36 per gut	30 per 12 guts	2.5 ± 1.98	57 per 24 guts	2.38 ± 1.66
Bagrid Catfish	50 per 12 guts	4.17 ± 2.25 per gut	19 per 12 guts	1.58. ± 1.24	69 per 24 guts	2.88 ± 2.11

### 3.3. Sizes of microplastics in water and sediments

**Fig 2:**
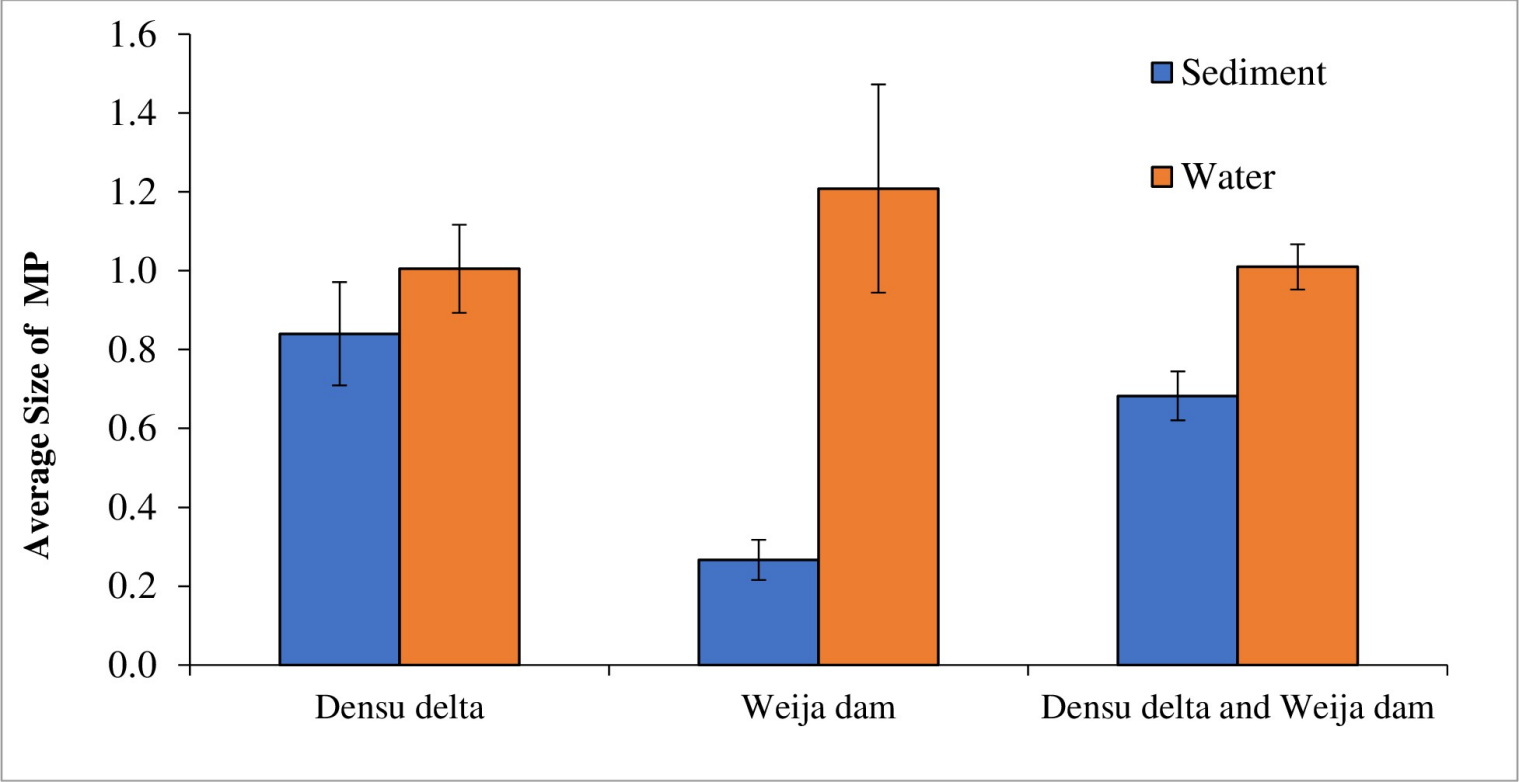
Size of microplastics in water and sediment of the Densu Delta and Weija Dam in Ghana.

The mean sizes of microplastics counted in the water and sediment in the study area are presented in Fig 2. In the Weija Dam, there was a significant (T = 4.888, P = 0.0003) difference in the mean sizes of microplastics between the water (1.208± 0.264) and sediment (0.267± 0.051) with about a 4.5-fold increase in the size of the microplastics in the water compared to the sediments (Fig 2). However, in the Densu Delta, there was no significant (T = 0.962, P = 0.349) difference in the size of microplastics in the sediment (0.840± 0.131) and water (1.005± 0.112) samples (Fig 2). A comparison of microplastics sizes in water samples from the Weija Dam (1.208± 0.264) and Densu Delta (1.005 ± 0.112) indicates no significant (T = 0.848, P = 0.411) difference (Fig 2). There was however a significant (T = 4.289, P = 0.0005) difference in the sizes of microplastics in Sediment from the Weija Dam (0.267± 0.051) and Densu Delta (0.840± 0.131) with a three-fold increase in the size of microplastics size in sediment from the Densu Delta compared to what is in the Weija Dam (Fig 2).

### 3.4. Number of microplastic particles in GI tracts of the fishes

**Fig 3:**
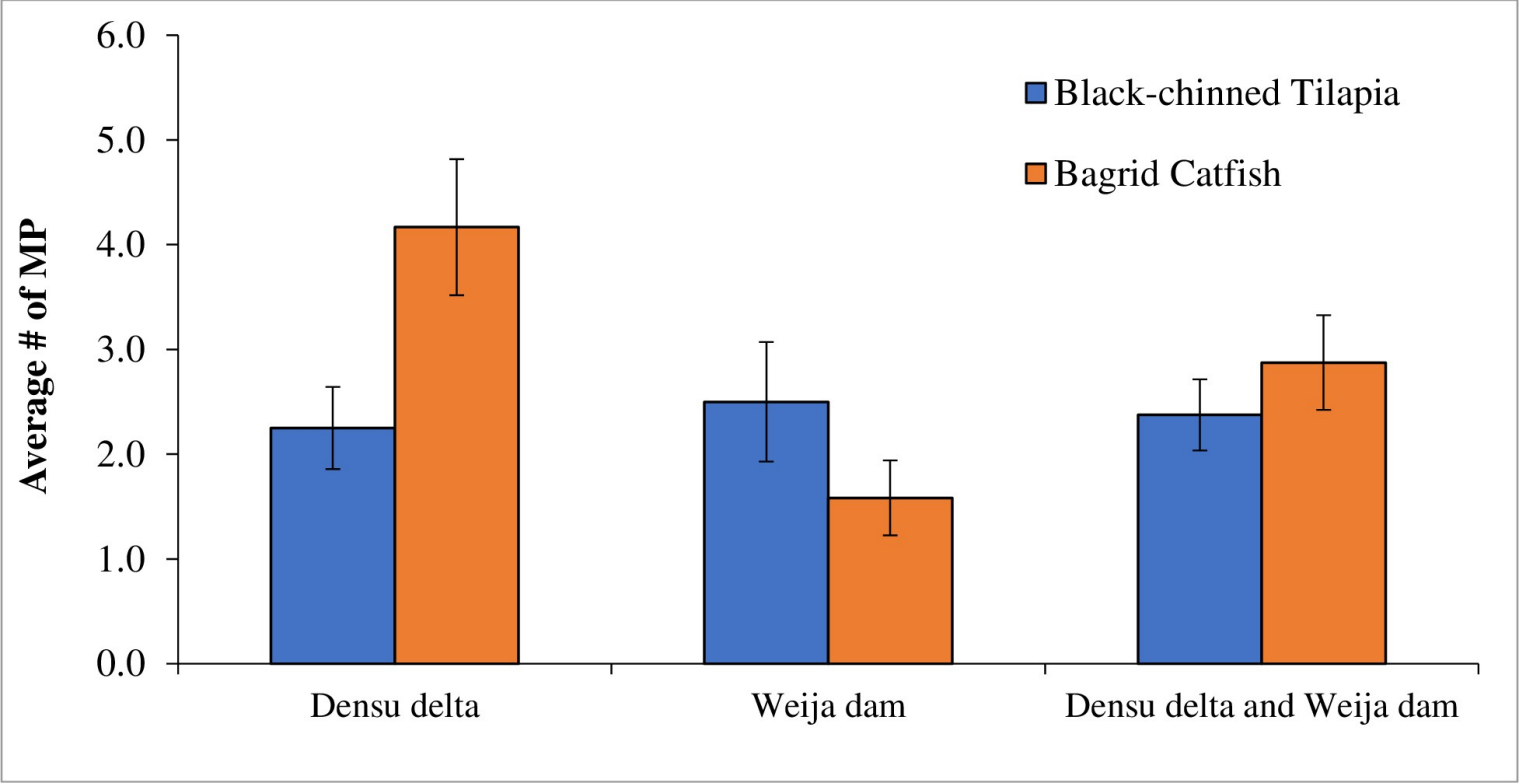
Number of microplastics in the gut of Black-chinned Tilapia and Bagrid Catfish in the Densu Delta and Weija Dam in Ghana.

The total numbers of microplastic particles in the Black-chinned Tilapia from Weija Dam and Densu Delta were 27 and 30 respectively as against 19 and 50 in the Bagrid Catfish respectively from Weija Dam and Densu Delta. In the Weija Dam, there was no significant (T = 1.36, P < 0.1874) difference in the mean number of microplastics in the GI tract of the Black-chinned Tilapia (2.50± 0.571) and Bagrid Catfish (1.583± 0.358) (Fig 3). However, in the Densu Delta, there was a significant (T = 2.53, P = 0.019) difference in the mean number of microplastics in the GI tract of the Black-chinned Tilapia (2.25± 0.392) and Bagrid Catfish (4.167± 0.649) with a two-fold increase in the number of MP in Bagrid Catfish compared to the Black-chinned Tilapia (Fig 3). A comparison of the number of microplastics in the GI tracts of Bagrid Catfish from the Weija Dam (1.583± 0.358) and Densu Delta (4.167± 0.649) indicates a significant (T = 3.484, P = 0.0021) difference (Fig 3). The Bagrid Catfish in the Densu Delta had a three-fold increase in microplastics than those in the Weija Dam. Also, there was no significant (T = 0.36123, P = 0.7214) difference in the number of microplastics in the GI tracts of Black-chinned Tilapia in the Weija Dam (2.50± 0.571) and Densu Delta (2.25± 0.392) (Fig 3). With respect to the pooled data from Weija and Densu there was no significant (T = 0.783, P < 0.438) difference in the number of microplastics in the GI tract of the Black-chinned Tilapia (2.375± 0.340) and Bagrid Catfish (2.875± 0.452).

### 3.5. Sizes of microplastics in GI tracts of the fishes

**Fig 4:**
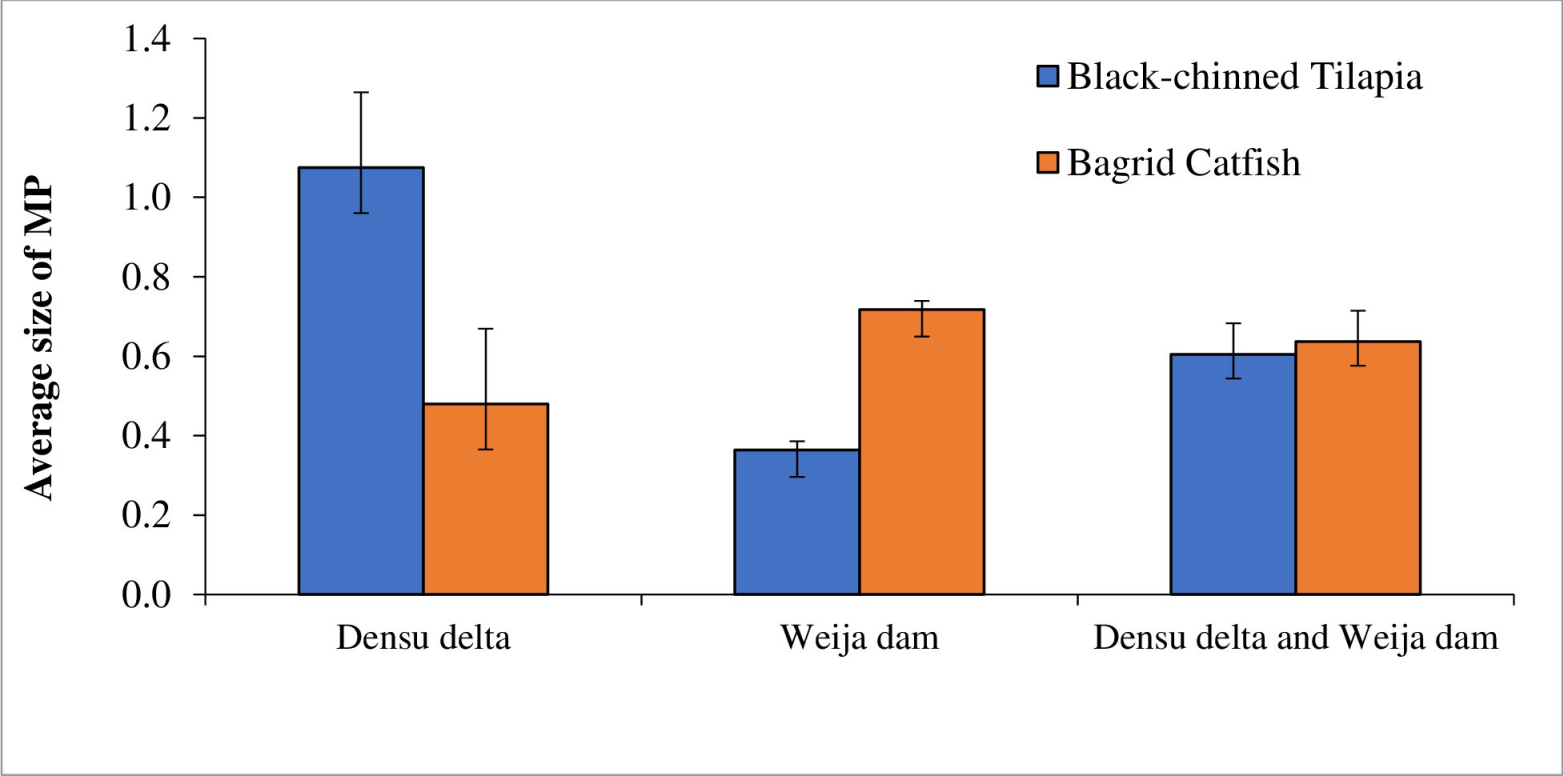
Sizes of microplastics in the gut of Black-chinned Tilapia and Bagrid Catfish in the Densu Delta and Weija Dam in Ghana.

The size of microplastics in the Black-chinned Tilapia at Weija Dam and Densu Delta ranged from 0.15 to 3.2 mm and 0.14 to 0.631 mm respectively. In the Bagrid Catfish, the sizes of microplastics ranged from 0.10 to 2.22 and 0.11 to 2.01 mm respectively from Weija Dam and Densu Delta. In the Weija Dam, there was a significant (T = 5.61, P < 0.00001) difference in the mean sizes of microplastics in the GI tracts of the Black-chinned Tilapia (0.364± 0.022) and Bagrid Catfish (0.718± 0.068) with a 1.4-fold increase in the size of microplastics in Bagrid Catfish compared to the Black-chinned Tilapia (Fig 4). Similarly, there was a significant (U = 99, P = 0.012) difference in the sizes of microplastics in the GI tracts of the Black-chinned Tilapia (1.075± 0.190) and Bagrid Catfish (0.480± 0.115) from the Densu Delta with about a two-fold increase in the size of microplastics in Black-chinned Tilapia compared to the Bagrid Catfish (Fig 4). A comparison of the sizes of microplastic in the GI tracts of the Bagrid Catfish in the Weija Dam (0.718± 0.068) and Densu Delta (0.480± 0.115) indicates a significant (U = 172, P = 0.0074) difference. The size of microplastics in the GI tracts of Bagrid Catfish in the Weija Dam on average was 1.5 larger than those in the Densu Delta. Similarly, the size of microplastics in the GI tracts of Black-chinned Tilapia in the Weija Dam (0.364± 0.022) and Densu Delta (1.075± 0.190) were significantly (T = 5.291, P = 0.00001) different with the sizes of microplastics in the Black-chinned Tilapia from the Densu Delta being approximately 1.6 times larger than those in the Weija Dam (Fig 4).

With respect to pooled data from Weija and Densu, there was a significant (U = 99, P = 0.0117) difference in the size of microplastics in the GI tract of the Black-chinned Tilapia (0.605± 0.078) and Bagrid Catfish (0.637± 0.061) with a 1.1-fold increase in the size of microplastics in Bagrid Catfish compared to the Black-chinned Tilapia.

### 3.6. Relationship between fish morphometrics and number of microplastics ingested

Based on pooled data for the Densu Delta and Weija dam, there was a weak positive correlation (R = 0.3) between the number of microplastics in the GI tracts and the weight of both Bagrid Catfish and Black-chinned Tilapia. There was also a weak positive correlation (R = 0.40) between the length of Black-chinned Tilapia and the number of microplastics ingested. There was a weak positive correlation (R = 0.35) between the number of microplastics and the weight of Black-chinned Tilapia. The low R values obtain however indicate no strong relationship between the number of microplastic ingested and the length and weight of both species of fish.

## 4. Discussion

### 4.1. General abundance of microplastic

Fish remains an important protein source for humans all over the world and contributes about 60% of Ghana’s animal protein intake [12]. Previous studies on microplastics contamination of fish in Ghana had focused largely on marine fisheries [13–15] with limited information on microplastics pollution of urban riverine systems. The study of microplastics in urban riverine ecosystems is particularly important because of their role in linking the terrestrial dump grounds of plastics to their final marine repository [13], their function of serving as habitats for fisheries [25], and the implication of these to human health from the consumption of these fisheries [11]. As far as we know this is the first study of microplastics pollution in urban riverine systems in Ghana and paves way for future studies in the West African sub-region where lack of capacity in institutions for carrying out microplastics research has resulted in the existence of huge data gaps [8].

We observed microplastics in all the environmental parameters studied namely sediment, water, Bagrid Catfish and Black-chinned Tilapia. Additionally, microplastics were found in all individuals of the two species of fish studied. These indicated that plastic pollution of the Densu River is widespread and may be exhibiting an impact on the purity of the river system as well as the security of the inhabiting organisms. There is also the potential that quantities of these microplastics are being ingested by humans who regularly consume these fishes.

Generally, the number of microplastics recorded per individual Bagrid Catfish (2.88 ±2.11) and Black-chinned Tilapia (2.38 ± 1.66) in this current study were lower than numbers of microplastics ingested per individual *Sardinella maderensis* (40 ± 3.8), *Dentex angolensis* (32 ± 2.7), *Sardinella aurita* (26 ± 1.6) from the nearshore and offshore areas of Coastal Ghana [15]. Data on the number of microplastics in sea waters and sediments of the nearshore and offshore areas of coastal Ghana were not readily available, but the relatively lower numbers of microplastics in the freshwater fishes compared to the marine fishes suggest plastic pollution and ingestion might be more severe in the marine environment than the freshwater ecosystems connecting the plastic sources in urban areas to the marine environment. It is worth noting that while there is a paucity of information and fragmentation of data on microplastics in freshwater ecosystems [26], plastic abundance in the oceanic gyres has often been reported to exceed that of zooplankton [27]. Thus, microplastics are ubiquitous but their prevalence from one environment to another is highly heterogeneous [28] and it appears plastic prevalence in the nearshore and offshore areas of Coastal Ghana are higher than the urban riverine system investigated in this study.

Despite the observation of the widespread nature of microplastic pollution in the Densu River, the condition factor (K) and growth coefficient (b) of the Black-chinned Tilapia generally indicated they were in good conditions and there was isometric growth. Although the Bagrid Catfish populations were in poor condition and indicated negative allometric growth, this study did not find any significant relationship between fish condition factor and microplastics ingestion and this was consistent with the findings of [15] in the Gulf of Guinea and [29] who reported no significant relationship between plastic ingestion and overall condition of various fish species from the North Sea.

According to [30], plastic pollution is more closely associated with sediments than water. Comparison of microplastics in environmental samples such as water and sediment are generally based on assumptions and cautions [14, 31, 32]. Although we did not compare microplastics abundance in the sediment directly with that of the water due to differences in the units of measurement, there were clear indications that microplastics abound in the sediment of the Densu River than the water on the assumption that one specific gravity of water is one gram per volume [14]. Based on this assumption, the microplastics abundance of 1 per 10 ml of water recorded for the Densu River in this study would translate to 1 per 10 g of water and thus less than the 3.88 microplastics per 10 g of sediment (Table 2).

The association of microplastics with sediment rather than water increases the potential of microplastics accumulation in filter-feeders and soil-dwelling biota compared to pelagic species [31] The pooled data from our study indicated that the Bagrid Catfish as a demersal omnivore ingested a higher number of microplastics compared to the pelagic omnivorous, Black-chinned Tilapia. The difference between the number of microplastic ingested by the two species of fish was however not statistically significant. The observation of a non-significant difference in the number of microplastics in the gut of the Bagrid Catfish and Black-chinned Tilapia was thus unexpected.

### 4.2. Spatial differences in microplastics abundance

Flow regimes and tidal energy flux are well-known hydrological phenomena that affect sedimentation and floatation of substances, and this could well influence the fate and distribution of microplastics in water bodies [33]. According to [34] vast quantities of plastics that should be detected floating on the sea surface are seemingly missing from the global budget and that investigation into the sinking properties of plastic is of paramount importance to modeling the fate of plastics. Also, large amounts of produced plastics are buoyant and should not sink on their own accord [35] but many low-density plastics rest on the seafloor and thus corroborating the existence of mechanisms that facilitate the sinking of plastic and the need for their investigation [36, 37].

Although the Densu Delta is a lotic system compared to the Weija Dam which is lentic, the mean number of microplastics in sediments of the two systems were statistically similar, just as mean numbers of microplastics in the water columns of the two systems were similar. These observations imply that the numbers of microplastics deposited in the two systems were not affected by the differences in their hydrology.

In contrast, our data indicated that the stagnant water system of the Dam promoted the floating of larger-sized microplastics while the flowing waters of the Delta did not show any selectivity in the deposition of microplastics between sediment and the water column. This is evident in the observation of a 4.5-fold significant increase in the size of microplastics in the Dam water compared to the sediments, the observation of no significant differences in the sizes of microplastics deposited in the Delta water and sediment, and the observation of significant larger sized microplastics in the sediment of the Delta compared to the Dam.

According to [38], bioturbation plays an important role in shaping the vertical distribution of microplastics. Additionally, biofouling, agglomeration with sediment particles and uptake into biological organisms are known factors that influence the vertical movement of microplastics between water columns and sediment [39]. The levels of bioturbation, biofouling and agglomeration in the lotic and lentic sections of the Densu River would not be the same and these need further investigations to ascertain the primary cause of the microplastics particle size segregation in the Dam.

This notwithstanding, disparities in the sizes of microplastics found in the sediment and water of the two systems did not reflect in the sizes of microplastics ingested by the inhabiting fishes as the sizes of microplastics in the gut of the two species did not follow any particular order. Thus, the sizes of microplastics in the gut of the Bagrid Catfish in the Dam were rather significantly larger than those in the Black-chinned Tilapia despite the demersal forager habits of the Bagrid Catfish while the sizes of microplastics in the gut of the Black-chinned Tilapia at the Delta were significantly larger than the Bagrid Catfish.

## 5. Conclusion

This study presented data on the occurrence of microplastics in water, sediment and two species of fish from the Densu River and examined microplastics distribution in the lentic and lotic sections of the river. Our results indicated widespread pollution of the Densu River with microplastics. Numbers of microplastics deposited in the Dam and Delta sections of the river were not affected by the differences in hydrology but the stagnant water system of the Dam promoted the floating of larger sized microplastics while the flowing waters of the Delta did not show any selectivity in the deposition of microplastics between sediment and the water column. The number of microplastics ingestions by the Bagrid Catfish was similar to the Black-chinned Tilapia but both species ingested a lower number of plastics than reported for marine fish species in coastal Ghana. We related this to the severity of microplastics pollution in the marine repositories of plastic than the freshwater riverine systems that connect them to the plastic sources in urban areas.
